# Exploring knowledge and management practices on ticks and tick-borne diseases among agro-pastoral communities in Southern Highlands, Tanzania

**DOI:** 10.14202/vetworld.2018.48-57

**Published:** 2018-01-21

**Authors:** Isack Ibrahim Kerario, Martin Simuunza, Emmanuel L. K. Laisser, Sebastian Chenyambuga

**Affiliations:** 1 Department of Disease Control, School of Veterinary Medicine, University of Zambia, P.O. Box 32379, Lusaka, Zambia; 2 Department of Animal, Aquaculture and Range Sciences, College of Agriculture, Sokoine University of Agriculture P.O. Box 3004, Morogoro, Tanzania; 3 School Quality Assurance Department, Eastern Zone, Ministry of Education, Science and Technology, P.O. Box 325, Morogoro, Tanzania

**Keywords:** acaricide, cattle, East Coast fever, indigenous knowledge

## Abstract

**Aim:**

The current study was conducted to assess the farmers’ knowledge and management practices on ticks and tick-borne diseases (TBDs) through individual interview using a structured questionnaire in Mbarali and Momba districts of Mbeya region.

**Materials and Methods:**

A total of 240 households, 120 from each district were asked to mention TBDs of cattle which they thought were the most important in their localities and period of the year when the diseases occurred more frequently. In addition, farmers were asked to describe clinical signs and management practices associated with the common TBDs that they knew.

**Results:**

The majority of respondents (46.2%) reported that East Coast fever (ECF) was the most important disease of cattle in the region, followed by anaplasmosis (33.8%), heartwater (15.4%), and babesiosis (4.6%). According to the farmers, ECF and anaplasmosis occurred more frequently during the dry season, while babesiosis and heartwater occurred more frequently during the rainy season. The majority of farmers were able to describe properly the signs of the common TBDs. Most farmers (80.4%) reported that they used acaricide to control ticks at a frequency of after every 2 weeks and a small proportion (15.8%) vaccinated their animals against ECF.

**Conclusion:**

It can be concluded that farmers in Mbeya have considerable knowledge on tick species and clinical signs of TBDs affecting their cattle. Based on the findings of the current study, it is recommended that integrated approach to the control of ticks and TBDs be adopted in the study area and many other areas that utilize agro-pastoral and pastoral cattle production systems.

## Introduction

Tanzania is endowed with a huge livestock resource due to the conducive climatic conditions that are suitable for livestock production. The country has a population of about 22.8 million cattle, 15.6 million goats, 35.5 million indigenous chickens, 24.5 million commercial chickens, and 2.01 million pigs [1]. Livestock is used for different functions such as ­the provision of essential food products, draft power, manure, and social functions. They also provide employment and income for the majority of the smallholder farmers living in the rural areas. Among the livestock species kept in the country, cattle have the greatest contribution to income, food and nutritional security for the rural communities [1]. Local breeds of cattle comprise 95% of the national herd, and the remaining 5% is made up of improved dairy and beef breeds, with the former being the main source of beef and other beef products in the country [1]. Traditional extensive farming is the main production system practiced by livestock keepers in Tanzania, whereby herds from different households intermingle during grazing in communal lands and watering points [2]. Under such production system, cattle are exposed to high risk of tick infestation leading to increased tick-borne diseases (TBDs) infection [2].

Ticks and TBDs constrain the improvement of livestock production efficiency in 11 countries of eastern, central, and southern Africa [2]. Tick-borne diseases cause high morbidity and mortality and lead to reduced growth rate, milk production, and fertility [2]. The most important TBDs of cattle include East Coast fever (ECF) (caused by *Theileria parva*), babesiosis (caused by *Babesia bigemina* and *Babesia bovis)*, anaplasmosis (caused by *Anaplasma marginale*), and heartwater (caused by *Ehrlichia ruminantium*) [3]. Moreover, these diseases cause a socioeconomic threat to the development of the livestock sector [2]. Studies have shown that ticks of the genera *Amblyomma* and *Rhipicephalus* are distributed in almost all areas in Tanzania, where cattle are kept and are the most important in the transmission of TBDs [4,5].

Prevailing methods for controlling TBDs include reducing tick infestations using acaricides, use of tick resistant breeds, immunization (vaccination), and treating infected animals by means of chemotherapy [5-7]. Acaricides are mainly used to control ticks in the tropics including Tanzania [2]. However, regular application of acaricides in Tanzania has not been sustainable probably due to their high costs. Acaricides are expensive for poor resourced farmers, and their intensive application on indigenous cattle is uneconomical [2]. Due to the high price of acaricide, most of the livestock keepers use sub-optimal concentrations on their animals [8].

Little research efforts have been performed to assess the indigenous knowledge and management practices of the traditional livestock keepers on the ticks and TBDs in Mbeya region of Tanzania. As such, there is limited information on the use of indigenous knowledge on ticks and TBDs, specifically in the southern highlands of Tanzania. Understanding indigenous knowledge and management practices of local livestock keepers on ticks and TBDs control is very important in the design and implementation of integrated disease control programs. The current study was conducted to assess the farmers’ knowledge and management practices on ticks and TBDs among livestock keepers in Mbarali and Momba districts of Mbeya region.

## Materials and Methods

### Ethical approval

Informed consent was obtained from all participants involved in this study.

### Study area

**Figure-1:**
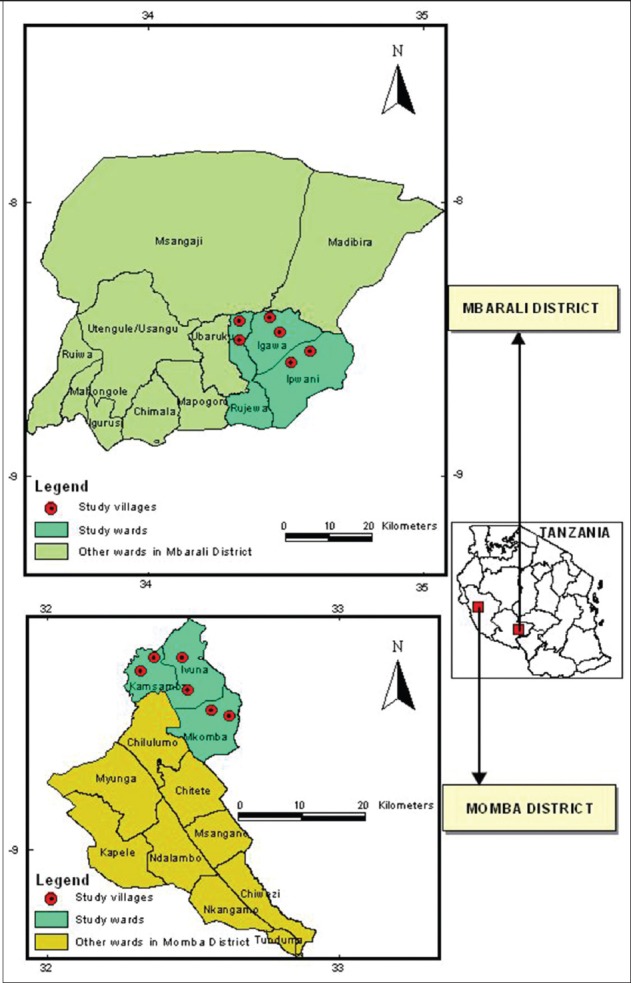
Map showing sampling districts, wards, and villages in Mbeya region.

The study was conducted in agro-pastoral communities of Mbarali and Momba districts of Mbeya Region in the Southern Highlands of Tanzania (Figure-1). The study was conducted with the full approval of households keeping cattle, district councils of the study areas, Sokoine University of Agriculture, and the University of Zambia. Mbeya region lies about 2400 m above sea level. The region has a subtropical climate with humid summers as well as dry winters. Average temperatures range from −6°C in the highlands to 29°C in the lowlands. The highest temperature is recorded in October and November while the lowest temperature is experienced in June and July. Mbeya has a unimodal type of rainfall, and the rains fall between October and May. The average rainfall per year is 900 mm. According to 2012 National census, the region had a human population of about 2,707,410. The main economic activity in the region is crop production, followed by livestock keeping. The dominant livestock species are cattle (911,889), followed by goats (275,659) [9]. Other livestock species of economic importance kept in the region include sheep, pigs, guinea fowls, ducks, geese, and rabbits. The region is divided into eight administrative districts, namely, Chunya, Ileje, Kyela, Mbarali, Mbeya, Mbozi, Momba, and Rungwe. In this study, sampling was performed in Mbarali and Momba districts. The two districts were selected based on their potentiality in having a large number of cattle.

### Sampling procedure

The purposive sampling procedure was employed to select districts. Selection of wards and villages in the district was performed using a simple random technique. Only villages that kept cattle were included in the sampling frame. In each of the two districts, three wards and two villages per ward were selected randomly, making a total of 6 wards and 12 villages. A total of 20 households in each village were randomly sampled, making a sample size of 120 in each district and 240 respondents for the total study. The heads of households were the main respondents. Where the main head of the household was not available, any senior member of the household was interviewed. Other members of the family were also allowed to provide supplementary information whenever needed. A structured questionnaire was used to collect information from each household. Information collected included household social economic characteristics, knowledge, perception and management practices on ticks and TBDs among livestock keepers in the study area.

### Statistical analysis

The collected information was initially entered into a Microsoft Excel spreadsheet before analysis. Data were analyzed using Statistical Package for the Social Sciences version 20 (SPSS Inc., USA). The Chi-square test was used to test for the association between categorical variables. All variables were considered significant at p≤0.05.

## Results

Results of the descriptive statistics of livestock keepers in Mbarali and Momba districts are presented in Table-1. The mean age of respondents in Mbararli and Momba districts was 45 (ranging from 28 to 89) and 44 (ranging from 19 to 81), respectively. However, the majority of farmers in the surveyed districts were between 31 and 45 years old. These comprised 52.5% and 45% of the respondents in Mbarali and Momba districts, respectively. Most of the respondents had experience of more than 10 years in livestock keeping. It was also observed that a large proportion of livestock keepers had primary school education in both districts. The majority of households in Mbarali (68.2%) and Momba (59.2%) districts reported that they kept between 10 and 50 cattle. It was also reported that a large percentage (52.5% in Mbarali and 46.7% in Momba) of livestock keepers owned a land size of 11-50 acres.

**Table 1:** Descriptive statistics of livestock keepers in Mbarali and Momba districts of Mbeya region.

Parameters	Respondents	Mbarali district (%)	Momba district (%)	p value
Age of household head (years)				
15-30	120	1.7	11.7	0.013
31-45	120	52.5	45	
46-60	120	35	29.2	
>60	120	10.8	14.2	
Experience of keeping cattle (years)				
<5	120	1.7	11.7	0.005
5-10	120	35	37.5	
>10	120	63.3	50.8	
Level of education of the household head				
No formal education	120	0.8	25	<0.0001
Primary education	120	80	65	
Secondary education	120	19.2	9.2	
College education	120	0	0.8	
Land owned by the household				
<5 acres	120	3.3	15.8	0.01
5-10 acres	120	36.7	34.2	
11-50 acres	120	57.5	46.7	
>50 acres	120	2.5	3.3	
Number of cattle raised by the household				
<10 cattle	120	3.3	34.2	<0.0001
10-50 cattle	120	68.3	59.2	
>50 cattle	120	28.3	6.7	

Percentage different across rows are significantly different (p≤0.05)

Farmers were asked to mention TBDs of cattle which they thought were important and period of the year when these diseases were reported to occur more frequently on cattle in their localities. The results of the outcome are presented in Table-2. ECF, anaplasmosis, babesiosis, and heartwater were reported to be important diseases of cattle in the study area. A large proportion of respondents (45.8%) in Mbarali reported that ECF was the most important tick-borne disease of cattle compared to others, followed by anaplasmosis (34.2%), babesiosis (11.7%), and heartwater (1.7%). In Momba, on the other hand, ECF was also reported by a large percentage (46.7%) of respondents to be the most important disease of cattle, followed by anaplasmosis (33.3%), heartwater (29.2%), and babesiosis (1.7%). When all data from the two districts were combined together (Table-2), ECF was still observed to be the most important disease of cattle in the region and was reported by 46.2% of the respondents, followed by anaplasmosis (33.8%), heartwater (15.4%), and finally babesiosis (4.6%).

**Table 2:** Tick-borne diseases of cattle and period of the year when they were reported to occur more frequently on cattle in Mbarali and Momba districts.

Tick-borne disease	Mbarali			Momba			p value	Overall		
		
	n	Occurrence	%	n	Occurrence	%		n	Occurrence	%
East Coast fever	120	Yes	45.8	120	Yes	46.7	0.102	240	Yes	46.2
		No	54.2		No	53.3			No	53.8
Anaplasmosis	120	Yes	34.2	120	Yes	33.3	0.108	240	Yes	33.8
		No	65.8		No	66.7			No	66.2
Babesiosis	120	Yes	11.7	120	Yes	1.7	0.001	240	Yes	4.6
		No	88.3		No	98.3			No	93.3
Heartwater	120	Yes	1.7	120	Yes	29.2	0.000	240	Yes	15.4
		No	98.3		No	70.8			No	84.6
East Coast fever	54	Rain season	57.4	57	Rain season	36.8	0.015	111	Rain season	46.8
	54	Dry season	42.6	57	Dry season	63.2		111	Dry season	53.2
Anaplasmosis	42	Rain season	45.2	39	Rain season	25.6	0.035	81	Rain season	35.8
	42	Dry season	54.8	39	Dry season	74.4		81	Dry season	64.2
Babesiosis	14	Rain season	71.4	2	Rain season	0.0	0.125	16	Rain season	62.5
	14	Dry season	28.6	2	Dry season	100.0		16	Dry season	37.5
Heartwater	2	Rain season	50.0	35	Rain season	94.3	0.153	37	Rain season	91.9
	2	Dry season	50.0	35	Dry season	5.7		37	Dry season	8.1

n=Number of respondents, percentage different across rows are significantly different (p<0.05)

Regarding the period of the year when TBDs were reported to occur more frequently in cattle, a larger proportion of farmers (57.4%) in Mbarali district reported that ECF occurred more frequently during the rainy season while in Momba the disease was reported to occur more frequently during the dry season (63.2%). With regard to anaplasmosis, many respondents reported that the disease occurred more frequently during the dry season in both Mbarali (54.8%) and Momba (74.4%) districts. It was observed that a large proportion of livestock keepers in Mbarali (71.4%) admitted that babesiosis occurred more frequently during the rainy season whereas in Momba the disease was reported to occur more frequently during the dry season. The majority of farmers (94.3%) reported that heartwater occurred more frequently during the rainy season. The overall assessment indicated that the majority of the farmers reported ECF and anaplasmosis to occur more frequently during the dry season, while babesiosis and heartwater were reported to occur more frequently during the rainy season.

Clinical signs of common TBD described by farmers in Mbarali and Momba districts are depicted in Table-3. When asked to describe the presentation of the most common tick-borne diseases, the majority of livestock farmers in both districts were able to describe properly the clinical signs associated with the common TBDs. For example, ECF was known as “ndigana kali” in Swahili language and was reported to be characterized by loss of appetite, fever, swollen lymph nodes, loss of conditions, labored breathing, and nasal discharge. Anaplasmosis was described by farmers as “ndigana baridi” and was reported to be characterized by loss of weight, anemia, fever, loss of milk production, and producing hard dung. Babesiosis was defined by farmers in Swahili as “Mkojo wa damu” meaning red urine and was characterized by fever, animals producing red urine and depression. Heartwater was described by farmers as “Moyo kujaa maji” in Swahili and was reported to be characterized by loss of appetite, circling or high stepping gait, fever, emaciation, loss of appetite, and hemorrhagic diarrhea.

**Table 3:** Clinical signs of common tick.borne diseases described by farmers in Mbarali and Momba districts.

Tick-borne disease	Clinical sign	n (%)		

		Mbarali	Momba	Overall
East Coast fever	Loss of appetite	44 (43.1)	13 (11.6)	57 (26.6)
	Fever	30 (29.4)	49 (43.8)	79 (36.9)
	Swelling of lymph nodes	23 (22.5)	46 (41.1)	69 (32.2)
	Loss of conditions	4 (3.9)	1 (0.9)	5 (2.3)
	Labored breathing	1 (1.0)	2 (1.8)	3 (1.4)
	Nasal discharge	0 (0.0)	1 (0.9)	1 (0.5)
Anaplasmosis	Loss of weight	28 (39.4)	7 (13.2)	35 (28.2)
	Anemia	15 (21.1)	5 (9.4)	20 (16.1)
	Fever	8 (11.3)	1 (1.9)	9 (7.3)
	Decreased milk production	12 (16.9)	0 (0.0)	12 (9.7)
	Producing hard dung	8 (11.3)	40 (75.5)	48 (38.7)
Babesiosis	Passing blood stained urine	14 (87.5)	2 (66.7)	16 (84.2)
	Fever	2 (12.5)	0 (0.0)	2 (10.5)
	Depression	0 (0.0)	1 (33.3)	1 (5.3)
Heartwater	Loss of appetite	2 (50.0)	5 (7.9)	7 (11.1)
	Emaciation	2 (50.0)	6 (10.2)	8 (12.7)
	Hemorrhagic diarrhea	0 (0.0)	6 (10.2)	6 (9.5)
	Fever	0 (0.0)	14 (23.7)	14 (22.2)
	High stepping gait	0 (0.0)	28 (47.5)	28 (44.4)

n=Number of responses

Table-4 summarizes season of the year when different types of ticks (brown year tick, blue tick, and bont tick) were most abundant. Brown ear tick (*Rhipicephalus appendiculatus*) was reported by the majority of farmers (61.5%) in Mbarali to be more abundant during the dry season (June to October) whereas in Momba, a larger proportion of farmers (94.4%) reported that the tick was more abundant during the rainy season (November to May). It was also observed that a large proportion of farmers in Mbarali (53.9%) and that of Momba (93.3%) reported that blue tick (*Boophilus* ticks) was most abundant during the rainy season. Bont tick (*Amblyomma* spp.) was reported by most of the respondents (68.1%) to be more abundant during the dry season in Mbarali while in Momba the majority of farmers (93.2%) mentioned that the tick was more abundant during the rainy season. In general, most of the ticks were reported to be more abundant during the rainy season (November to May) in the region (Table-4).

**Table 4:** Period of the year when ticks are reported to occur more frequently on cattle in Mbarali and Momba districts.

Season	Brown ear tick, Frequency (%)			Blue tick, Frequency (%)			Bont tick, Frequency (%)			Overall ticks, Frequency (%)	
			
	Mbarali	Momba	p value	Mbarali	Momba	p value	Mbarali	Momba	p value	Mbarali	Momba
Rain season	32 (27.4)	102 (94.4)	<0.0001	63 (53.9)	98 (93.3)	<0.0001	30 (25.6)	96 (93.2)	<0.0001	38 (32.2)	96 (88.9)
Dry season	72 (61.5)	2 (1.9)		44 (37.6)	2 (1.9)		82 (68.1)	2 (1.9)		44 (37.3)	0 (0)
All seasons	13 (11.1)	4 (3.7)		10 (8.6)	5 (4.8)		5 (4.3)	5 (4.9)		36 (30.5)	12 (11.1)

Percentage different across rows are significantly different (p<0.05)

Three control practices of ticks and TBDs (no tick control, acaricide application, and vaccination against ECF) were reported in the study area (Table-5). In Mbarali larger proportion of farmers (73.3%) used acaricide to control ticks and few of them vaccinated their cattle against ECF (15.8%). However, vaccination against ECF was only reported in this district. In Momba large proportion of farmers (87.5%) also reported that they used acaricide to control ticks whereas a small proportion of them (12.5%) did not control ticks on their cattle. Two methods (dipping and hand spray) of acaricide application were reported in the study area as depicted in Table-5. In Mbarali both methods were practiced, with dipping (72.9%) being the most common method of acaricide application used in the district. Hand spraying was the only method used to control ticks in Momba district and was practiced by 100.0% of the farmers. The overall assessment showed that hand spraying was the most common method of acaricide application reported by 63.2% of farmers in the region. Besides, the majority of farmers in Mbarali reported that there were dip tanks in their village, whereas in Momba all the farmers interviewed stated that there were no dip tanks in their villages. Moreover, most farmers in Mbarali reported that the available dip tanks were in good condition and all of them were operating. For frequency of acaricide application, four practices were reported in the study area, namely, weekly, biweekly, monthly, and occasionally. In Mbarali district, the majority (95.1%) of the farmers reported that they controlled ticks after every 2 weeks, and the remaining (4.9%) controlled ticks on a monthly basis. In Momba, 51.0% of farmers controlled ticks biweekly, whereas the remaining 39.2%, 6.9%, and 2.9% controlled ticks on monthly, weekly, and occasionally, respectively. When all data combined most farmers (73.0%) showed that they controlled tick after every 2 weeks.

**Table 5:** Tick and TBD control practices reported by farmers in Mbarali and Momba districts.

Parameters	Mbarali n (%)	Momba n (%)	p value	Over all n (%)
Tick control practice				
No tick control	13 (10.8)	15 (12.5)	0.002	28 (11.7)
Acaricide application	88 (73.3)	105 (87.5)		193 (80.4)
ECF vaccination	19 (15.8)	0 (0.0)		19 (15.8)
Method of applying acaricide				
Dipping	78 (72.9)	0 (0.0)	0.000	78 (36.8)
Hand spraying	29 (27.1)	105 (100.0)		134 (63.2)
Frequency of applying acaricide				
Weekly	0 (0.0)	7 (6.9)	0.000	7 (3.4)
Biweekly	97 (95.1)	52 (51.0)		149 (73.0)
Monthly	5 (4.9)	40 (39.2)		45 (22.1)
Occasionally	0 (0.0)	3 (2.9)		3 (1.5)
Brand name of acaricide used				
Albadip 10% EC (Alphacypermethrin)	26 (24.3)	5 (4.8)	0.000	31 (14.6)
Alfanex 10% EC (Alphacypermethrin)	43 (40.2)	0 (0.0)		43 (20.3)
Cybadip 15 EC (Cypermethrin)	0 (0.0)	10 (9.5)		10 (4.7)
Twigatraz 12.5 EC (Amitraz)	3 (2.8)	0 (0.0)		3 (1.4)
TikTik 12.5 EC (Amitraz)	0 (0.0)	5 (4.8)		5 (2.4)
Paranex 100 EC (Alphacypermethrin)	35 (32.7)	85 (81.0)		120 (56.6)
Drugs used to treat ECF				
Butalex	22 (34.9)	1 (1.8)	0.000	23 (19.2)
Parvexon	12 (19.0)	45 (78.9)		57 (47.5)
Oxytetracycline 20%	29 (46.0)	11 (19.3)		40 (33.3)
Drugs used to treat anaplasmosis				
Oxytetracycline 20%	41 (100.0)	40 (100.0)	N/A	81 (100.0)
Drugs used to treat babesiosis				
Oxytetracycline 20%	0 (0.0)	12 (100.0)	N/A	12 (100.0)
Drugs used to treat heartwater				
Oxytetracycline 20%	2 (100.0)	35 (100.0)	N/A	37 (100.0)
Access to veterinary services				
Livestock extension officer	79 (92.9)	3 (2.8)	0.000	82 (42.3)
Selfadministration	6 (7.1)	106 (97.2)		112 (57.7)

n=Number of respondents, percentage different across rows are significantly different (p<0.05). TBD=Tick borne diseases, ECF=East Coast fever

Farmers who were applying acaricides by hand spraying were able to tell the brands and price of each brand (Table-5). The brands of acaricides mentioned by farmers included Albadip 10% (Alphacypermethrin, Bajuta International, Limited, Tanzania), Alfanex 10% EC (Cypermethrin, Ronheam international Co. Limited, Tanzania), Twigatraz 12.5 EC (Amitraz, Twiga Chemical Industries (T) Ltd), TikTik 12.5EC (Amitraz, Farm Base Limited, Tanzania), and Paranex 100 EC (Alphacypermethrin, Farm Base Limited, Tanzania). In Mbarali most farmers (40.2%) reported that they used Alfanex to control ticks, while in Momba most farmers (81.0%) said they used Paranex to control ticks. In totality, the majority of farmers (56.6%) reported that they used Paranex to control ticks in the region.

Regarding the treatment of TBDs, 92.9% of respondents in Mbarali district reported that they sought assistance from government veterinarians or livestock extension officers for the treatment of their animals, while in Momba 97.2% of farmers stated that they bought drugs and administer by themselves (Table-5). Those who treated the animals by themselves, when asked about the type of drug they use, majority of them mentioned oxytetracycline (OTC) 20% was the drug used to treat ECF, anaplasmosis, babesiosis, and heartwater. Other drugs pointed out were Butalex and Parvexon for treatment of ECF. In Mbarali most farmers used OTC 20% to treat their animals against ECF while in Momba the majority of respondents said that they used Parvexon to treat their animals against ECF. In general, most farmers reported that they used Parvexon to treat ECF (Table-5).

## Discussion

The current study explored knowledge and management practices on ticks and TBDs among of livestock keepers in Mbarali and Momba districts of Mbeya region. Socioeconomic characteristics of livestock keepers are also reported in this study. The age of most farmers was within the common labor force group in Tanzania. People in this group are quite active, creative and participate in various social and economic activities [10]. Our findings are in agreement with that reported by Mwambene *et al*. [11] in Njombe, Mufindi, Muheza, and Bagamoyo districts of Tanzania. Furthermore, household education and experience in keeping livestock reported in the study are in agreement with those reported by other researchers somewhere else [12-14]. In particular, the majority of the household heads attained the formal elementary education and had enough experience in livestock keeping. This implies that the farmers included in this study had reasonable knowledge and skills regarding livestock diseases and could describe pertinent information on how to manage and control them. According to Nkonya *et al*. [15], in any rural community, education is an important tool for socioeconomic development as it provides better prospects to access information, goods, and services and enables farmers to undertake appropriate actions. The observed high number of cattle per household mirrors the importance of cattle to the livelihood of pastoral and agro-pastoral communities. This is in agreement with the report by MLFD [1] that indigenous cattle are the major source of meat and milk in Tanzania and offer a genetic resource base which is abundantly available and can be exploited for improving the well-being of rural people.

Among the TBDs of cattle, ECF was mentioned by the majority of farmers as an important disease in Mbarali and Momba districts compared to other TBDs. Similar findings have been reported in Uganda [16] that ECF was the most prevalent cattle disease, followed by trypanosomiasis. Another study conducted by Laisser *et al*. [14] reported foot-and-mouth disease as the most important disease in Tarime, Serengeti, Meatu, and Maswa districts of Tanzania, followed by babesiosis. In general, the majority of farmers mentioned ECF to be the most important disease of cattle, followed by anaplasmosis, heartwater, and babesiosis. In Tanzania, among the TBDs ECF has been reported to be the most important disease of cattle [2,6,17]. The disease has been reported in the country to be the major cause of deaths, especially among calves, causing calf mortality of about 40-80% [18].

The present study found that in general, the livestock farmers were knowledgeable about ticks and TBDs. However, there was some dissimilarity between the occurrence of the diseases and the extent of exposure to tick infestation in their responses. For example, farmers in Mbarali reported that ECF was more prevalent during the rainy season, but the brown ear tick (*R. appendiculatus*) which is the vector of *T. parva*, the causative agent of ECF, was reported to be more abundant during the dry season [1]. On the other hand, in Momba district ECF was reported to be more prevalent during the dry season while the brown ear tick was reported to be more abundant during the rainy season. These results are similar to those reported by Chenyambuga *et al*. [12] and Laisser *et al*. [14]. In general, in the current study, the farmers were knowledgeable on ticks and TBDs, some differences among the districts are influenced by geographical location and the extent of exposure to tick infestation. The farmers’ opinion that ticks (*R. appendiculatus*) are most common during the dry season is due to the fact that during the rainy season farmers have other farm activities, hence being unable to make close observations to their animals. However, brown ear ticks are usually abundant during the rainy season due to favorable conditions for tick multiplication [7]. According to Walker *et al*. [19], the pattern of seasonal occurrence of *R. appendiculatus* is controlled by the unfed adults that enter diapause of the behavioral type and as a result, do not engage in host-seeking until the rains start. In the southern parts of Africa including southern highlands of Tanzania where there is a well distinct season, there is an obvious relationship between the commencement of the rains and adult tick activity. In these areas, only one generation of ticks (*R. appendiculatus*) per year is observed. In hot regions including Mwanza in Tanzania, where rain falls all over the year, adults may be seen all year round. In these areas, several overlapping generations can be completed annually, and there is no clear pattern of seasonal abundance of ticks [19]. On the other hand, babesiosis and heartwater were reported to be more prevalent during the rainy season in the region. The observed higher prevalence could be associated with a high abundance of tick vectors (blue ticks and bont ticks) reported during the rainy season.

The majority of farmers had adequate knowledge of the symptoms of the different TBDs affecting their animals through extension services provided by the government village extension officers.

In pastoral and agro-pastoral communities, knowledge and skills on livestock management are commonly transferred from fathers to children starting from a young age, such that when one grows up, he would able to take care of the herd. According to Catley [20], pastoralists have superior diagnostic skills for animal diseases which are orally passed on from one generation to the next, particularly from the elders to the young ones. Most of the symptoms mentioned by livestock keepers concur with those described in the literature. For example, Bock *et al*. [3] describe clinical signs of babesiosis as fever, inappetence, depression, increased respiratory rate, weakness, and reluctance to move. Other symptoms of babesiosis include hemoglobinuria (red urine) and development of anemia and jaundice. The main symptoms of ECF as described by Gwamaka *et al*. [21] include fever, swelling of lymph nodes, loss of appetite, excessive salivation and lacrimation, staring hair coat, dry muzzle, dropped ears, ruminal stasis, anorexia, and dyspnea. Anaplasmosis, the disease caused by *A. marginale* is characterized by progressive hemolytic anemia associated with fever, weight loss, abortion, and decreased milk production [21]. Heartwater (caused by *E. ruminantium*) is characterized by sudden onset of fever, tachycardia, inappetence, hyperesthesia, high-stepping gait, twitching eyelids, chewing, and hemorrhagic diarrhea [21]. The observation in the current study agrees with other studies [12,14] who reported that pastoralists and agro-pastoralists have adequate diagnostic skills for animal diseases which conform to the veterinarian diagnosis criteria. In the current study, OTC was the common drug used to treat ECF, anaplasmosis, babesiosis, and heartwater. In addition to OTC other drugs such as butalex and parvexon were also used to treat ECF. This implies that the farmers in the study area have adequate enough knowledge on treating TBDs.

The majority of respondents stated that they used acaricide to control ticks on their cattle. Our findings concur with that reported by Swai *et al*. [8] in pastoral community of Ngorongoro district, who reported that over 50% of livestock keepers used acaricide for tick control. Our observation also agrees with that reported by Chenyambuga *et al*. [12] who reported that 59.2% of livestock keepers around Lake Victoria basin applied acaricide to control ticks and TBDs. The main methods of acaricide application in the study area were by hand spraying and dipping. However, most of the farmers in this study reportedly used hand spraying to control ticks. This is in consistence with the observation by Mugisha *et al*. [16] that hand spraying was the most preferred method of acaricide application in pastoral and agro-pastoral communities. This is because most farmers believe that hand spraying was cheap, convenient, and effective [12]. Despite this method being easy to use, it is not a good method to control ticks in communal areas as some farmers do not use correct dilution dose of acaricides, while others apply occasionally [8]. Indiscriminate and improper dilution of acaricides can lead to the development of tick resistance [8]. Moreover, when using spray method, the acaricides do not reach all parts of the animal body; hence, not all ticks are killed [4,8]. It is recommended that where animals from different households graze together dipping should be used for applying acaricides as it is more efficient and reliable in controlling ticks compared to hand spraying [4]. Hand spraying can be chosen if and only if farmers have been trained to prepare the correct dilution of the acaricide and the sites for tick attachment on the body of the animal where spraying should be done. Dipping was not normally used in the study area especially in Momba district because the dips were not available in the surveyed villages at the time of conducting this study.

In this study, the majority of farmers were spraying/dipping their animals after every 2 weeks. Application of acaricide by dipping animals once in 2 weeks in pastoral and agro-pastoral communities had been reported to be economical as it reduces costs of acaricide and limits the losses caused the death of the animals due to TBDs [22]. However, it is imperative for livestock extension officers to consider farmers experience before implementing any strict tick control system. For example, in Serengeti district of Mara region, it was discovered that some of the wards where strict tick control measure was implemented, the levels of tick infestation declined, but increased the incidences of animals to succumb from *T. parva* infection and ECF compared to the wards in which there was no strict dipping/spraying regime [4].

Another TBDs control practice implemented in the study area was vaccination against ECF by infection and treatment method which was only practiced in Mbarali district and only by a small proportion of farmers implying that most farmers are not aware or there is limited access to ECF vaccine in the study area. In Tanzania among the TBDs, ECF is the number one killer causing a large number of cattle deaths [2]. Vaccination against ECF offers an overlook of less costly and more effective control of the diseases without continued dependence on expensive acaricides [6]. The goodness of ECF vaccination is that it increases the survival rate of calves and reduces mortality rate down to 2% annually among the pastoralists. Immunization has been reported to be associated with a reduction in the development of tick resistance to acaricides due to reduced frequency of using acaricides, as well as to lower tick control costs by up to 50% [23]. In addition, the majority of smallholder dairy farmers cut acaricide use by more than three quarters with a subsequent reduction in costs to livestock keepers and less environmental pollution.

## Conclusion

Tick-borne diseases cause significant morbidity and mortality of cattle in the southern highlands of Tanzania. Among the TBDs, ECF is considered as the main disease of cattle while babesiosis is regarded as a minor problem in Mbarali and Momba districts of Mbeya region. The majority of farmers in Mbarali and Momba districts have adequate knowledge of the symptoms of the different TBDs. Based on the findings of the current study, different strategic planning and cost-effective control measures should be implemented based on the magnitude of TBDs in the region to reduce morbidity and mortality caused by different TBDs in the study area. Further study is recommended to determine the prevalence of TBDs and the associated risk factors in the study area.

## Authors’ Contributions

This study was conceived and designed by Isack Ibrahim Kerario (IIK), Martin Simuunza (MS), Emmanuel L.K. Laisser (ELKL), and Sebastian Chenyambuga (SC). IIK and ELKL performed field data collection. Data analysis was performed by IIK. IIK, MS, ELKL, and SC participated in drafting and editing of the manuscript, and all authors read and approved the final version of the manuscript.
